# Posttraumatic growth and recovery from addiction

**DOI:** 10.1080/20008198.2017.1369832

**Published:** 2017-09-13

**Authors:** Eyal Haroosh, Sara Freedman

**Affiliations:** ^a^ School of Social Work, Bar Ilan University, Ramat Gan, Israel

**Keywords:** Addiction-related growth, recovery, social support

## Abstract

**Background**: It is well documented that individuals coping with adverse events report both negative outcomes, such as posttraumatic stress symptoms, as well as positive changes, described as posttraumatic growth. Positive changes are also reported in people who have recovered from substance abuse. It seems plausible from the literature that both of these types of positive changes have elements in common. To date, no published studies have examined positive outcomes among people who have recovered from addiction.

**Objectives**: In this study, posttraumatic growth in individuals who were formerly addicted to alcohol or substances, termed ‘addiction-related growth,’ was examined. Addiction-related growth refers to the growth that an individual undergoes as a result of the addiction itself, and the recovery from the addiction. A successful recovery from addiction is associated with positive changes, particularly regarding spirituality and meaning-making, and the construct of addiction-related growth may explain why.

**Method**: This cross-sectional study examined growth among 104 individuals who had recovered from addiction who were recruited from addiction treatment programmes, between February and July 2012. Questionnaires assessed demographics and substance abuse use and treatment, posttraumatic growth (PTGI); social support (Perceived Social Support Questionnaire); and help-seeking (*Willingness to Seek Help Scale*). Data was analysed using an analysis of variance (ANOVA), Pearson correlations, and multiple regression.

**Results**: Results indicated that addiction-related growth is a phenomenon that accurately captures the positive changes experienced as a result of an individual’s struggle with addiction and recovery. This growth was found to be associated with participation in 12-steps programmes, and to be predicted by levels of perceived social support.

**Conclusions**: The results show that recovery from addiction is associated with addiction-related growth. These positive changes, along with the importance of communal social support, resemble the changes that take place as a result of processes described in 12-steps programmes.

## Abstract (es)

**Planteamiento**: Está bien documentado que los individuos que se enfrentan a acontecimientos adversos indican tanto resultados negativos –tales como síntomas de estrés postraumático– como cambios positivos descritos como crecimiento postraumático. También se indican cambios positivos en personas que se han recuperado del abuso de sustancias. A partir de la literatura, parece plausible que ambos tipos de cambios positivos tengan elementos en común. Hasta la fecha, ningún estudio publicado ha examinado resultados positivos entre las personas que se han recuperado de una adicción.

**Objetivos**: En este estudio, se examinó el crecimiento postraumático, denominado ‘crecimiento relacionado con la adicción’, en individuos que antes eran adictos al alcohol o a otras sustancias. El crecimiento relacionado con la adicción se refiere al crecimiento que un individuo experimenta como resultado de la adicción en sí, y de recuperarse de la adicción. El éxito de la recuperación de la adicción se asocia con cambios positivos, particularmente en lo que respecta a la espiritualidad y a dotar de significado, y el constructo del crecimiento relacionado con la adicción puede explicar por qué.

**Método**: Este estudio transversal examinó el crecimiento en 104 individuos que se habían recuperado de una adicción, que fueron reclutados de los programas de tratamiento de adicciones, entre febrero y julio de 2012. Los cuestionarios evaluaron la demografía, y el abuso de sustancias y su tratamiento; el crecimiento postraumático (PTGI); el apoyo social (Cuestionario de apoyo social percibido, o *Perceived Social Support Questionnaire*); y la búsqueda de ayuda (Escala de disposición para buscar ayuda o *Willingness to Seek Help Scale*). Los datos se analizaron mediante un análisis de varianza (ANOVA), correlaciones de Pearson y regresión múltiple.

**Resultados**: Los resultados indicaron que el crecimiento relacionado con la adicción es un fenómeno que capta con precisión los cambios positivos experimentados como resultado de la lucha de un individuo con la adicción y la recuperación. Se encontró que este crecimiento estaba asociado con la participación en programas de doce pasos y que se predecía por los niveles de apoyo social percibido.

**Conclusiones**: Los resultados muestran que la recuperación de la adicción está asociada con el crecimiento relacionado con la adicción. Estos cambios positivos, junto con la importancia del apoyo social comunitario, se asemejan a los cambios que se producen como resultado de los procesos descritos en los programas de doce pasos.

## Abstract (zh)

**背景：**大量文献显示个体在应对负性事件时既报告负面结果（例如创伤后应激症状），也报告积极改变，称之为创伤后成长。从物质滥用中恢复的人也报告了积极改变；从文献看来这两种积极改变似乎是有共同的要素。迄今为止，还没有已经发表的研究考察过从成瘾中恢复给人带来的积极结果。

**目标：**在本研究中考察曾经的酒精或者物质成瘾者的创伤后成长（称为‘成瘾相关的成长’）。‘成瘾相关的成长’指成瘾本身和成瘾恢复过程给个体带来的成长。成功的恢复和积极改变相关联，特别是精神性和意义建构。成瘾相关的成长的结构可能可以解释其中的原因。

**方法：**本横断研究在2012年2月到7月之间，从成瘾治疗项目中招募了104个成瘾恢复者考察其成长。问卷询问了人口统计资料和物质滥用以及治疗情况，创伤后成长（PTGI），社会支持（主观社会支持问卷），和帮助寻求（帮助寻求意愿量表）。使用方差分析（ANOVA）、皮尔逊相关分析和多元回归对数据进行统计分析。

**结果：**结果说明，‘成瘾相关的成长’这种现象可以准确描述个体在戒瘾和恢复过程后出现的积极改变。这种成长和参与‘十二阶段（12-steps）’项目有关，可以被主观社会支持的水平所预测。

**结论：**结果显示出成瘾恢复和‘成瘾相关的成长’有关联。这些积极改变以及公共社会支持的重要性，都和 ‘十二阶段’项目的过程引起的改变相似。

## 1. Introduction

Positive psychology emphasizes the adaptive ways in which individuals interact with their environment (Seligman & Csikszentmihalyi, 2000), and a growing literature has in recent years examined the positive impact of coping with extremely difficult events. Studies have shown that 30–70% of individuals report positive outcomes subsequent to a traumatic event (Linley & Joseph, 2004). Posttraumatic growth (PTG) is a result of the process of change that takes place in the aftermath of challenging events (Tedeschi & Calhoun, 2004) and has been reported after traumatic events such as sexual assault (Grubaugh & Resick, 2007), medical conditions such as cancer or HIV, and even significant positive life events, such as becoming a parent (Ben-Ari, Shlomo, Sivan, & Dolizki, 2009).

A separate body of studies regarding addiction have shown that traumatic events are related to the use of drugs, the likelihood of becoming dependent on them, and the process of recovery from addiction. Before, during, and after the use of drugs (i.e. in recovery), individuals are likely to experience many potentially traumatic events. In addition, the struggle with recovery is in and of itself a daunting challenge (Fullilove et al., 1993).

However, some of the literature on substance abuse pertains to the positive changes wrought by this abuse. As studies have shown that a feeling of existential emptiness may contribute to the initial use of drugs (Roos, Kirouac, Pearson, Fink, & Witkiewitz, 2015), it is perhaps not surprising that increased faith and spiritual levels have been shown to be related to one’s *recovery* from substance abuse (Jarusiewicz, 1999). Some substance abuse treatment programmes, particularly the 12-steps programmes, emphasize many aspects of personal growth, such as the need for faith (Minehan, Newcomb, & Galaif, 2000) and the adoption of daily spiritual activities. These are seen as allowing the individual to disengage from any discomfort (Dermatis & Galanter, 2016). From this perspective, recovery is seen as requiring the adoption of a new way of life that is meant to sustain this recovery, manifested in changes in lifestyle, habits, values, and gradual spiritual growth. It should also be said that social support has been found to be essential for recovery, particularly from individuals who themselves are recovered from former addiction (Subbaraman, Kaskutas, & Zemore, 2011). Social support therefore should be defined as including both perceived social support – on the individual level – as well as active giving support to others and receiving support from others – on the group level. This latter type of support, that is typical in substance abuse programmes, is perhaps an example of communal coping, where a group or community recognizes a problem as a shared issue that requires joint coping (Rossetto, 2015).

The changes that an individual needs to make during recovery, in terms of spirituality and values, echo PTG. The process of change, via the individual’s receipt of social support from others who have experienced similar challenges, is also reminiscent of the PTG model. As such, it seems likely that PTG might be a relevant concept for those recovering from substance abuse. As far as the authors of the current study are aware, only one unpublished study has examined posttraumatic growth among this population. In that study, individuals who had recovered from addiction were shown to have rates of PTG (or, as they termed it, addiction-related growth) that are similar to those of people who experienced traumatic events (Washton, 2007).

The current study attempts to further the knowledge in this area by way of examining PTG as well as two other factors that are related both to successful recovery and to PTG – social support and willingness to seek help (Bischof, Rumpf, Meyer, Hapke, & John, 2007; Grella & Stein, 2013) – among a population of people who had recovered from addiction. The study also assesses whether participation in 12-steps programmes, with their emphasis on spiritual change, is related to PTG. The study hypothesized that increased participation in NA/AA (measured by frequency of attendance, 12-steps programme participation, and work as a sponsor), as well as length of abstinence, social support, and willingness to seek help would be related to higher levels of PTG.

## 2. Method

### 2.1. Participants

Subjects were 104 individuals who were formerly addicted who were currently participating in addiction treatment programmes. These programmes help individuals whose primary issue is addiction; individuals who have comorbidity with a psychiatric diagnosis were excluded as they are treated in different centres. Inclusion criteria were attendance at one of these treatment programmes. The subjects included 89 men (86%), 11 women (11%), and four individuals (3.8%) who did not specify their gender. Twenty-six (25%) were married, 33 (31.7%) were single, 37(35.6%) were divorced, and three (2.9%) were widowed or separated. Subjects’ ages ranged from 21 to 62 years of age (mean age 40.2). All subjects were Israeli nationals, with 52.8% born in Israel; the remainder were immigrants, with 34.6% born in the former Soviet Union, 3.8% born in Europe or America, and 3.8% born in Asia and Africa.

### 2.2. Data collection

The study was approved by the Ethics Committee of the School of Social Work, Bar-Ilan University and the Division of Research of the Ministry of Social Affairs, and was conducted between February and July 2012. The researcher (EH) contacted administrators at public health service addiction treatment centres throughout Israel, and received permission to carry out the study. Questionnaires were then distributed to participants in a group setting.

### 2.3. Materials

Measures were administered either in Hebrew or Russian, depending on the participant’s first language. All questionnaires were translated into Hebrew and Russian, and back-translated.

#### 2.3.1. Demographic and background information

Information regarding gender, age, marital status, and birthplace was gathered. In addition, items covered substance use, addiction recovery, treatment, and level of involvement in Narcotics Anonymous/Alcoholics Anonymous (NA/AA). Substance abuse was assessed by asking participants which substances they had used that impacted them. Recovery was assessed by asking participants about time periods they considered themselves clean. Both these variables were assessed subjectively.

##### 2.3.1.1. *Willingness to seek help scale (Cohen, 2016*)

This 30-item questionnaire consists of three parts: the individual’s recognition of the existence of a problem s/he cannot cope with alone; his/her willingness to share this problem with another person; his/her willingness to hand over some of the control of his/her life to someone else. Questions are scored on a 4-point score, 0 to 3. Cronbach’s alpha for the current study was 0.75.

##### 2.3.1.2. *Perceived social support questionnaire (Zimet, Dahlem, Zimet, & Farley, 1988*)

This 12-item questionnaire examines the perception of social support relating to three sources of support: family, friends, and significant others. Items are answered on a 7-point scale, 1 to 7. Cronbach’s alpha for the current study was 0.805.

##### 2.3.1.3. *Posttraumatic growth inventory (PTGI, Tedeschi & Calhoun, 1996*)

This is a 21-item questionnaire relating to the change experienced by a person since the time of the traumatic event, and is divided into five dimensions: (1) a recognition of new possibilities in life and changing priorities; (2) changing relationships with others; (3) attributing meaning to the personal strength of the body; (4) personal and spiritual development, and (5) a greater appreciation of life. Questions are answered on a 6-point scale, 1 to 6. Cronbach’s alpha for the current study was 0.92.

### 2.4. Data analysis

Statistical analyses were performed using SPSS software, version No. 21. Differences between groups were calculated using analysis of variance (ANOVA). Relationships between the study variables were calculated using Pearson correlations. The relative contribution of each variable to explain the variance of posttraumatic growth was calculated using a stepwise multiple regression.

## 3. Results

### 3.1. Substance abuse

As can be seen in Table 1, a wide range of substances had been abused, with most participants reporting their use of more than one substance. In addition, the majority had participated in NA or AA, two to three times per week. Table 1 also presents statistics regarding recovery from substance abuse. Half of the participants had participated in a 12-steps programme, although most had not been sponsors. The majority reported the current period of being drug-free as the first significant period of remission.

**Table 1.** Substance abuse and recovery.

		*N* (%)
Substance	Alcohol	67 (66.3)
	Marijuana	75 (74.3)
	LSD and/or Ecstasy	53 (51)
	Sleeping pills	41 (40.6)
	Energy pills	36 (35.6)
	Heroin, opium and coke	66 (65.3)
	Cocaine and crack	60 (60.6)
	Other	49 (49.5)
Participated in NA or AA	Yes	85 (81.7)
Frequency of participation in NA or AA	Once a month	8 (9.8)
	Once a fortnight	20 (24.4)
	2–3 times per week	42 (51.2)
	Almost every day	12 (14.6)
12-steps programme	Yes	51 (50)
Sponsor	Yes	22 (21.4)
	No	81 (78.6)
Significant period/s of being clean	0	2 (2)
	First	59 (59.6)
	Second	22 (22.2)
	Third	9 (9.1)
	Four or more	7 (7.1)

Correlations between study variables are shown in Table 2; as can be seen, PTG (*M *= 99.88, *SD *= 17.57) was significantly correlated with frequency of NA/AA attendance (*M *= 2.24, *SD *= 1.28; *p *= .002), help-seeking, (*M *= 1.81, *SD *= 0.29, *p *= .011), and social support (*M *= 4.91, *SD *= 1.06, *p *= .00) but not length of abstinence.

**Table 2.** Correlations.

	PTGI	Abstinence length	NA.AA frequency	Help seeking	Social support
PTGI	1	.125	.313^a^	.259^b^	.467^a^
Abstinence length		1	−.001	−.060	.035
NA AA frequency			1	.169	.231^b^
Help seeking				1	.244^b^
Social support					1

^a^Correlation is significant at the 0.01 level (2-tailed). ^b^Correlation is significant at the 0.05 level (2-tailed).

An analysis of variance compared those who had participated in a 12-steps programme (*N* = 50) with those who had not (*N* = 50). Participants in 12-steps programmes had been abstinent significantly longer, *F*(1,98) = 7.70, *p *= .007, ηp 2 = .07; reported significantly higher social support, *F*(1,90) = 7.005, *p *= .01, ηp 2 = .06; and showed higher PTG in the new possibilities subscale, *F*(1,85) = 6.59, *p *= .01, ηp 2 = .07, and the spiritual change subscale, *F*(1,91) = 11.85, *p *= .001, ηp 2 = .11.

Participants who had served as a sponsor (*N* = 20) were compared with those who had not served as a sponsor (*N* = 81) in regard to length of abstinence, PTG subscales, help-seeking, and social support. A one-way ANOVA showed that sponsors, as compared with those who had not taken on this role, had been abstinent significantly longer, *F*(1,99) = 32.92, *p *= .00, ηp 2 = .25, and reported significantly higher levels of PTG in three subscales: personal strength, *F*(1,914) = 4.1, *p *= .04, ηp 2 = .04; spiritual change, *F*(1,92) = 6.29, *p *= .01, ηp 2 = .06; and appreciation of life, *F*(1,92) = 7.94, *p *= .006, ηp 2 = .07.

To test the hypothesis that the total level of PTG was a function of frequency of NA/AA meeting attendance, 12-steps programme participation, acting as a sponsor, length of abstinence, help-seeking, and social support, a multiple regression analysis was performed. The results of the regression indicated that social support explained 21.8% of the variance in PTG (adj R2 = .218, *F*(6,79) = 4.9, *p* < .01; beta = 0.4, *p* < .01), no other variables were significant.

## 4. Discussion

This study examined posttraumatic growth in treatment seeking patients who had been addicted to alcohol or substances. The results support the existence of addiction-related growth, replicating the findings of the one previous study that was conducted on this subject (Washton, 2007). Although growth has often been found to occur in the aftermath of various traumatic, stressful, and life-changing events, the results of the current study suggest that addiction recovery might be an additional antecedent for such growth.

The path to recovery following addiction has been thoroughly studied and described, and successful recovery from addictions has been found to be associated with changes in priorities and increased self-awareness (McMillen, Howard, Nower, & Chung, 2001). Participation in self-help groups includes a number of processes and mechanisms of change that support the individual while s/he remains drug-free, and the intent of these processes is to assist the individual in maintaining his/her personal growth. Twelve-steps programmes emphasize the belief that recovery from addiction is a spiritual journey.

This study showed that greater frequency of participation in self-help addiction-recovery programmes was significantly correlated with PTG levels. Participants in 12-steps programmes reported a more successful recovery – in terms of length of abstinence – as well as higher levels of spiritual growth and the ability to recognize and consider new possibilities in life. Acting as a sponsor was also related to greater length of abstinence, spiritual growth, personal strength, and appreciation of life. Taken together, these findings confirm the study hypotheses and demonstrate that greater participation in self-help programmes, with their emphasis on growth, is related to greater PTG.

When an individual enters a self-help programme, s/he is by definition seeking help, and indeed help-seeking is seen as a key element – perhaps even the initial element – of these programmes. Seeking help indicates a readiness to accept assistance and a basic commitment to undergo change; by contrast, a reluctance to seek help is interpreted as a wish to deny the existence of a problem and a lack of motivation to change (Grella & Stein, 2013). In this study, help-seeking was positively correlated with PTG.

The major finding of this study regards the relationship found between social support and posttraumatic growth: i.e. social support increases as PTG increases, and it is the only significant predictor of PTG. Social support for traumatized individuals, particularly from other individuals who have experienced similar traumatic events, is a key concept in PTG development, and there is clear evidence that it helps mitigate the negative effects of traumatic events (Andrews, Brewin, & Rose, 2003). In line with these findings, social support has been shown to be related to PTG among cancer patients (Schroevers, Helgeson, Sanderman, & Ranchor, 2010) and among child survivors of World War II (Lev-Wiesel & Amir, 2003). The results reported here highlight another aspect of social support: communal support. Instead of understanding social support as something that happens on an individual level, communal support emphasizes the importance of conceptualizing a problem as something shared by a group or community, which is tackled by the group; communal support is a key feature of substance abuse programmes. Communal support has been shown to be related to levels of posttraumatic growth in flood survivors (Wlodarczyk et al., 2016), and individuals who survived armed conflict and participated to communal rites showed lower levels of PTSD and higher levels of growth (Gasparre, Bosco, & Bellelli, 2010).

As in the treatment of post-trauma victims, social support is also a key element in addiction treatment programmes. Individuals who are addicted come to understand that ‘loneliness is an enemy,’ and learn to give up the addictive substance in favour of intimacy, intimate interpersonal relationships, and trust (Ronel, 1995). Help giving is as important as receiving help. In summary, the findings indicate that addiction-related growth is one of the positive effects of recovery from addiction. The findings also reinforce the idea that social support is crucial in the process of withdrawal and recovery from substance abuse. It seems that these positive processes, long recognized by participants in 12-steps programmes, have a theoretical foundation in posttraumatic growth.

This study has several limitations. The research tools used may not have been optimal: the willingness to seek help scale is long, and it was difficult for the participants to complete. Additional information regarding background variables were omitted. In addition, the sample was a convenience sample, and was made up of a large preponderance of individuals who took part in NA/AA programmes and came from public care settings. As both groups receive a great deal of social support, in comparison with others recovering from addiction, the results found here should be replicated among a more heterogeneous group.

A third limitation is related to the reliability of research findings. Like many other studies on posttraumatic growth (Nishi, Matsuoka, & Kim, 2010), this study is based solely on self-report questionnaires and – as self-reports do not take into account subjects’ wish to please – the results may not reflect the reality of the situation in its entirety. Lastly, additional information regarding the subjects’ backgrounds, such as length of residence in Israel and social economic status, were not collected. Future studies would be well advised to consider the use of objective measures, such as urine tests, as well as staff evaluations.

## 5. Conclusions

This study indicates that although addiction, and recovery from addiction, are processes that are related to adversity, they can also result in positive psychological outcomes. Addiction-related growth as a concept allows for a better understanding of these positive changes, and how they are related to social support and treatment type. Finally, 12-steps programmes may yield positive results, due to their emphasis on social support.

## Highlights

- Posttraumatic growth follows negative experiences
- Positive outcomes have been described in recovery of substance abuse
- Posttraumatic growth has not been previously examined in individuals who have recovered from addiction
- These results show that posttraumatic growth is a relevant concept for individuals who have recovered from addiction
- Social support is related to levels of posttraumatic growth in individuals who have recovered from addiction
